# Serum Zinc Level in Children with Febrile Seizure

**Published:** 2020

**Authors:** Firozeh HOSSEINI, Ali NIKKHAH, Mojdeh AFKHAMI GOLI

**Affiliations:** 1 Pediatric Neurology, Hamedan University of Medical Sciences, Hamedan, Iran,; 2 Pediatric Neurology, Research Center, Shahid Beheshti University of Medical Sciences, Tehran, Iran.; 3 Department of Social Medicine, School of Medicine, Hamedan University of Medical Sciences, Hamedan, Iran

**Keywords:** Febrile seizure, Zinc, Trace element, Children

## Abstract

**Objectives:**

Febrile seizure (FS) is the most common type of seizure in children. FS is a genetic age-limited seizure, which only occurs with febrile illness. Today, it is known that genetic factors play a major role in the occurrence of FS. Nevertheless, some trace elements, such as zinc, may play an important role in the occurrence of FS. In this study, we investigated the serum level of zinc in patients with FS and febrile children without seizure (control group).

**Materials &Methods:**

This prospective case-control study was conducted on 41 patients with simple and complex FS as the case group and 41 febrile children without seizure as the age- and sex-matched control group. The participants were admitted to Best Hospital of Hamadan, Iran between January 2013 and January 2014 . The children’s age ranged from six months to five years. Serum zinc levels were measured by atomic absorption spectroscopy in these groups.

**Results:**

The mean serum zinc levels were 70.41±20.46 and 92.73±17.62 mcg/dL in the case and control groups, respectively (P=0.001). The results showed that the serum zinc level in children with FS was significantly lower than that of the control group.

**Conclusion:**

Based on the present results, serum zinc level was lower in children with febrile seizure. However, further basic research is needed to examine the efficacy of zinc supplements in the prevention of FS.

## Introduction

Febrile seizure (FS), previously known as febrile convulsion, is the most common seizure in children (1, 2). FS usually occurs between six months and five years of age (3). It is a genetic age-limited disorder, which only occurs with febrile illness (4). It is important to exclude central nervous system (CNS) infections and electrolyte imbalance before FS diagnosis. Also, patients should have no history of afebrile seizures (4, 5). FS is classified into two simple and complex groups. Simple FS is generalized, lasts for 10-15 minutes, and occurs once in 24 hours. Conversely, complex FS is characterized by prolonged focal seizures, which occur more than once in 24 hours (5). 

The main mechanism of FS pathophysiology is not clear yet (2, 5). Today, it is known that genetic factors play a major role in the occurrence of FS, although some environmental factors, such as trace elements (e.g., zinc), may be involved in the association of genetic changes with FS occurrence (5, 6). Generally, zinc is an important trace element, which contributes to growth and development, neurological function, nerve impulse transmission, and hormone release (7). It also stimulates the activity of pyridoxal kinase, as the enzyme modulating the level of gamma aminobutyric acid (GABA) (8). In this prospective study, we evaluated zinc level in children withthe first FS attack and febrile children without seizure. Our findings can help clinicians make a definite decision about the use of zinc supplements for preventing the recurrence of febrile seizures via regulation of some neurological functions (e.g., reducing neuronal excitability and affecting synaptic vesicles) (6, 9). 

## Materials &Methods

This prospective case-control study was performed on 41 patients with their first FS attack (simple or complex) as the case group and 41 febrile children without seizure as the age- and sex-matched control group. The participants were admitted to Besat Hospital of Hamadan, Iran between January 2013 and January 2014. Generally, FS is an age-limited fever-induced seizure in children, which occurs between three months and six years of age (especially six months to five years) without CNS infection, electrolyte imbalance, or previous afebrile seizure. As mentioned earlier, simple FS is a brief (<10-15 min) generalized seizure, which occurs once in 24 hours. Conversely, complex FS is a focal seizure, which lasts longer than 15 minutes and occurs more than once in 24 hours.

The exclusion criteria were as follows: history of congenital structural anomalies of CNS; developmental delay; history of seizure with or without fever; history of prematurity and neonatal seizure; CNS infection; electrolyte abnormalities; any known conditions associated with zinc deficiency (e.g., acrodermatitis enteropathica); and consumption of zinc supplements. Within two to four hours of hospitalization, venous blood samples were collected for serum zinc measurements. Serum zinc level was measured by atomic absorption spectroscopy. The normal range of zinc in the serum was 70-110 mcg/dL, and zinc levels below 40 mcg/dL were indicative of zinc deficiency. 

Data were analyzed using t-test, Chi-square, ANOVA, and Kolmogorov-Smirnov test in SPSS version 18. Quantitative variables, such as age, were analyzed by measuring mean and standard deviation (SD). P-value less than 0.05 was considered statistically significant. This study was approved by the Ethics Committee of Hamadan University of Medical Sciences. 

## Results

A total of 82 children (41 patients per group) were evaluated in this study. In the case group, 23 children were male (53.3%), and 18 children (46.7%) were female. In the control group 22 children were male (43.3%) and 19 children were female (56.7%). The age of all participants was between six months and five years. The mean age of subjects in the FS and control groups was 33.1±14 and 31.5±7 months, respectively. 

In the case group, 28 patients had simple FS, and 13 patients had complex FS. Maximum and minimum levels of serum zinc were 115 and 43 mcg/dL in the case group, respectively. Also, maximum and minimum levels of serum zinc in the control group were 125 and 65 mcg/dL, respectively. The mean serum zinc level was 70.41±20.46 mcg/dL in the case group and 92.73±17.62 mcg/dL in the control group; there was a significant difference between the groups (P=0.001). The results showed that the serum zinc level in patients with FS was obviously lower than that of febrile children without seizure (Table 1). 

In this study, none of children were zinc-deficient (<40 mcg/dL) in the two groups. In the case group, the mean serum zinc level was 70.96±22.95 mcg/dL in patients with simple FS and 69.23 ±14.44 mcg/dL in patients with complex FS; there was no significant difference between these patients(P=0.804).

**Table 1:** Demographic characteristics and brief results of children evaluated in this study

Mean (SD) serum zinc level (mcg/dL)^*^	Mean (SD) age (months)	Male/female	Groups
70.41±20.46	33.1±14	23/18	Case (n=41)
92.73±17.62	31.5±7	22/19	Control (n=41)

*P=0.001

## Discussion

FS is the most common seizure in children around the world. Children with FS are usually between six months and five years old. The main pathogenesis of FS is still unclear, although some factors may play an important role in children with genetic predisposition to FS (9, 10). It is known that zinc is a co-factor of glutamic acid decarboxylase, which modulates the production of gamma aminobutyric acid (GABA) in CNS. It modulates the activity of glutamic acid decarboxylase, which is a rate-limiting enzyme in the synthesis of GABA. Also, it increases the affinity of neurotransmitters such as glutamate to their receptors and facilitates the inhibitory effect of calcium on NMDA receptors (8, 11). 

In the present study, the mean serum zinc level was 70.41±20.46 mcg/dL in the case group and 92.73±17.62 mcg/dL in the control group. There was a significant difference between the groups (P=0.001), although none of our participants were zinc-deficient (<40 mcg/dL). In a similar case-control study from Iran, Salehiomran et al. revealed that the mean serum level of zinc in FS patients was significantly lower than that of febrile children without seizure (58 vs. 71 mcg/dL) (11). Moreover, in a previous case-control study, the mean serum level of zinc in children with FS was lower than that of controls; however, the difference was not statistically significant (52.8 vs. 56.1 mcg/dL) (P=0.66) (13). 

In another similar study, Heydarian et al. showed that the mean serum level of zinc was lower in patients with simple FS, compared to febrile age-matched children without seizure, and the difference was statistically significant (14). In another recent study, Lee et al. showed that the mean serum level of zinc in children with FS was significantly lower than that of afebrile children with seizure (15). Furthermore, Ehsanipour et al. reported lower serum levels of zinc in children with FS, compared to the control group (children with fever and children with afebrile seizures) (16). In another study from India, the mean serum level of zinc was lower in children with febrile seizures, compared to febrile children without seizure (17).

The results of most studies in this area are somewhat similar to our study. However, there are few studies reporting inconsistent results. Cho et al. showed that there was no significant difference in the serum zinc level of children with FS and control children (18). Similarly, Kafadar et al. showed no significant difference in the serum zinc level of children with febrile convulsions and afebrile healthy children (19). Moreover, in the most recent study from Iran, researchers not only found no significant difference in the serum level of zinc between children with FS and healthy children, but also the mean serum level of zinc in the FS group was scarcely higher than that of the control group (20). 

Finally, some limitations must be acknowledged in this study, such as the small sample size and lack of zinc level measurements in the cerebrospinal fluid. Also, it is still unclear if lower zinc level in some febrile children is related to seizure occurrence.

**In Conclusion, **In this study, we found that the serum level of zinc in children with FS was lower than the control group (febrile children without seizure), and the difference was statistically significant. However, we cannot recommend zinc administration for prevention of FS, and further basic research is necessary in the future. 
